# Immobilization of Graphene Oxide on the Permeate Side of a Membrane Distillation Membrane to Enhance Flux

**DOI:** 10.3390/membranes8030063

**Published:** 2018-08-15

**Authors:** Worawit Intrchom, Sagar Roy, Madihah Saud Humoud, Somenath Mitra

**Affiliations:** Department of Chemistry and Environmental Science, New Jersey Institute of Technology, Newark, NJ 07102, USA; wi6@njit.edu (W.I.); sagar@njit.edu (S.R.); msh36@njit.edu (M.S.H.)

**Keywords:** graphene oxide, membrane distillation, flux, desalination, mass transfer coefficient

## Abstract

In this paper, a facile fabrication of enhanced direct contact membrane distillation membrane via immobilization of the hydrophilic graphene oxide (GO) on the permeate side (GOIM-P) of a commercial polypropylene supported polytetrafluoroethylene (PTFE) membrane is presented. The permeate side hydrophilicity of the membrane was modified by immobilizing the GO to facilitate fast condensation and the withdrawal of the permeate water vapors. The water vapor flux was found to be as high as 64.5 kg/m^2^·h at 80 °C, which is 15% higher than the unmodified membrane at a feed salt concentration of 10,000 ppm. The mass transfer coefficient was observed 6.2 × 10^−7^ kg/m^2^·s·Pa at 60 °C and 200 mL/min flow rate in the GOIM-P.

## 1. Introduction

The demand for clean water has considerably increased around the world and is becoming a critical issue due to an increasing population and growing industrialization [1]. The consumption in industrial sector alone was up by more than 200% in 2015 when compared to 1995 [2]. The amount of freshwater in the world is limited and much of it is polluted, and consequently brackish or sea water are attractive sources of pure water [3]. However, energy efficient and cost effective desalination processes are important for that to happen. There are two most popular techniques for desalination, namely reverse osmosis (RO) and multi-stage flash (MSF) distillation [4,5]. While these are well established techniques that have much merit, they face limitations, such as high energy consumption, fouling, and high capital investment [6]. As a result, alternative desalination technologies, including solar evaporation and membrane distillation (MD), are being explored [4,7].

In the MD process, a hot feed is used to generate a vapor pressure gradient across the porous hydrophobic membrane and the vapors are condensed in a cold permeate [8,9,10]. The process can be conducted at a relatively low temperature (50–90 °C), hence waste heat, solar energy, or geothermal energy can be utilized as heat sources for heating the brine [11,12,13,14,15]. Other advantages include high rejection of the dissolved non-volatile species, able to handle highly concentrated brine with less fouling, low operating pressure, and less space requirement when compared to MSF [8,16,17,18]. However, MD still is faced with some barriers, such as relatively low water vapor flux in comparison with other conventional systems, flux reduction due to temperature and concentration polarization, pore wetting and membrane fouling, and lack of high-efficiency membranes [19,20].

Several hydrophobic polymers, including polytetrafluoroethylene (PTFE), polypropylene (PP), and polyvinylidene difluoride (PVDF) have been utilized as membrane materials [9,21,22,23], and different methods have been employed for the synthesis and modification of these membranes to improve the performances [24,25,26,27,28,29,30,31,32,33,34,35,36]. Nanotechnology has enabled the development of advanced membranes based separation techniques [18,24,26,29,31,32,33,34,35,37,38,39]. Nanomaterials (NMs), such as Fe_3_O_4_, TiO_2_, SiO_2_, carbon nanotubes (CNTs), nanodiamonds (NDs), and graphene oxide (GO) have been incorporated via blending or coating method to improve the membrane efficiency [10,18,24,32,33,34,35,39,40,41,42], and different functionalized CNTs were used to fabricate a bilayered structures that have shown significant enhancement in flux [32]. Recently, GO has found a niche in membrane separations [43,44]. The GO is comprised of highly oxidized graphene sheet having various functional groups, including hydroxyl, carbonyl, and epoxy groups on its surface. These functional groups minimize the aggregation of GO in dispersion state, provide reaction moieties and make GO hydrophilic [45]. Recent studies have shown that GO could potentially improve the mechanical properties as well as selectivity, antifouling, and the permeate flux [33,46,47,48].

In MD, membrane itself plays the crucial role in enhancing the flux and selectivity. While most of the researches have focused on different membrane modifications [24,27,28,30,33,48], our previous studies have shown that permeate side hydrophilization of the membrane can significantly improve the water vapor permeation rate [16]. The rapid water vapor removal from the permeate side boundary layer is one of the most important consideration in increasing the concentration gradient for enhanced mass transfer. However, hydrophilization using strong oxidizing agents are quite hazardous and tend to reduce the mechanical strength of the membrane. An approach that involves NM modification by immobilizing hydrophilic NMs has the advantage of improving membrane characteristics as well as easy adjustment. GO can be significantly hydrophilic with high oxygen content and it is conceivable that the hydrophilicity of the permeate side can be improved by the incorporation of GO. In our previous study, the immobilization of the GO in the feed side have shown significant enhancement in flux [42]. There, the GO was instrumental in enhancing the partition coefficient and permeation of water vapor from the feed side. In the present study, we present a complimentary approach where the GO is immobilized on the permeate side. Here, the GO enhances the overall flux by providing sites for condensation of the permeated water vapor, which facilitates the rapid removal of water, thus enhancing overall mass transport.

## 2. Materials and Methods

### 2.1. Materials

Sodium chloride (NaCl), acetone, GO sheet (42–52% carbon), and polyvinylidene difluoride (PVDF) powder (Mw ~ 500 K) were purchased from Sigma–Aldrich (St. Louis, MO, USA). Deionized water was used in all experiments. The membrane that was used in the MD experiments was flat composite PTFE membrane supported with polypropylene nonwoven fabric (Advantec MFS, Dublin, CA, USA; 129 μm thick, 0.2 µm pore size and 70% porosity).

### 2.2. Experimental Procedure

**Figure 1:**
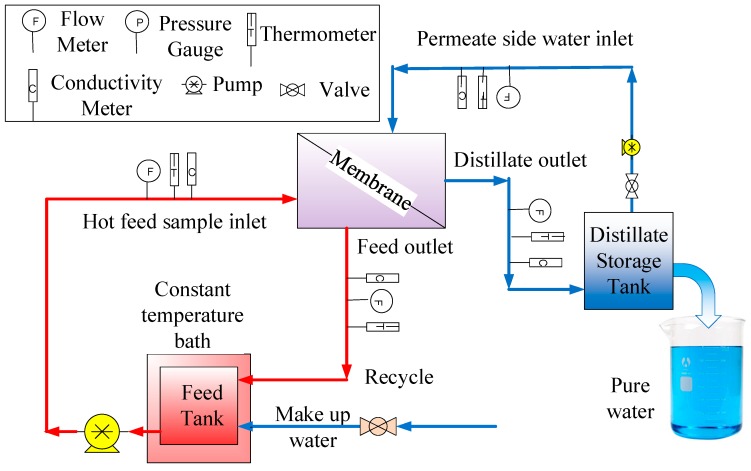
Schematic diagram of the experimental set up for direct contact membrane distillation (DCMD) application.

MD experiments were conducted in the direct contact MD (DCMD) configuration. Figure 1 shows the schematic diagram of the MD system used in the laboratory. The system consists of a DCMD cell and PTFE membrane with an effective contact area of 11.94 cm^2^. The feed and permeate flow were regulated by peristaltic pumps (MasterFlex Easy Load, Cole-Parmer, Vernon, IL, USA). The hot aqueous NaCl solution at different concentrations was passed through the feed side of the membrane in the DCMD cell and the cold distilled water was pumped through the permeate side of the membrane. Additional hot water was supplied to the feed water reservoir throughout the experiment to maintain the concentration constant. A counter current flow mode was used for feed and permeate water flow through the module. The constant temperature water bath (Neslab Water Bath Model GP 200, NESLAB Instruments, Inc., Newington, NH, USA) was used to maintain constant feed temperature, and the permeate temperature around 18 °C was controlled by a bench top chiller (Polyscience LS5, Cole-Parmer, USA). Temperatures of feed and permeate side were monitored by temperature sensors (Four-channel Data Logging Thermometer, RS-232, Cole-Parmer, USA). The experiment was repeated for three times and less than 1% relative standard deviation was observed.

### 2.3. Fabrication of GOIM-P

In fabrication of the graphene oxide immobilized membrane on the permeate side (GOIM-P), the uniform dispersion of GO in the organic solvent and immobilization of GO on the membrane surfaces are considered the most important steps during membrane fabrication. Ten mg of GO was added to 8 g of acetone and sonicated for 10 h to ensure the uniform dispersal of GO into the organic solvent. 0.2 mg of PVDF was separately dissolved in 2 g of acetone and the PVDF solution was finally mixed with GO suspension. The mixed PVDF-GO suspension was then cast drop wise slowly and uniformly on the permeate side of the membrane to immobilize the GO on the surface. After that, the immobilized membrane was rinsed with extra acetone to remove excess PVDF from the membrane pores and the surface.

### 2.4. Characterization of GOIM-P

A scanning electron microscopy (SEM, Model LEO 1530, Carl Zeiss SMT AG Company, Oberkochen, Germany) was used to characterize the morphology of the fabricated GOIM-P. The samples for SEM was prepared by cutting the membranes into a square of 0.5 cm × 0.5 cm, placing on a specimen stub followed by carbon coating. The GOIM-P was further illustrated by Raman spectroscopy (DXR Raman microscope, Thermo Scientific, Waltham, MA, USA). The thermal stability of GOIM-P was investigated by thermal gravitational analysis (TGA 8000, Thermogravimetric analyzer, PerkinElmer, Waltham, MA, USA). The contact angles measurements were used to study the hydrophobicity/hydrophilicity of the permeate surface while using an Attension apparatus (model Theta, Biolin Scientific UK, Manchester, UK). The water drop method on dry membrane was employed and five measurements were taken to obtain the average value.

## 3. Results and Discussion

### 3.1. Characterization of GOIM-P

**Figure 2:**
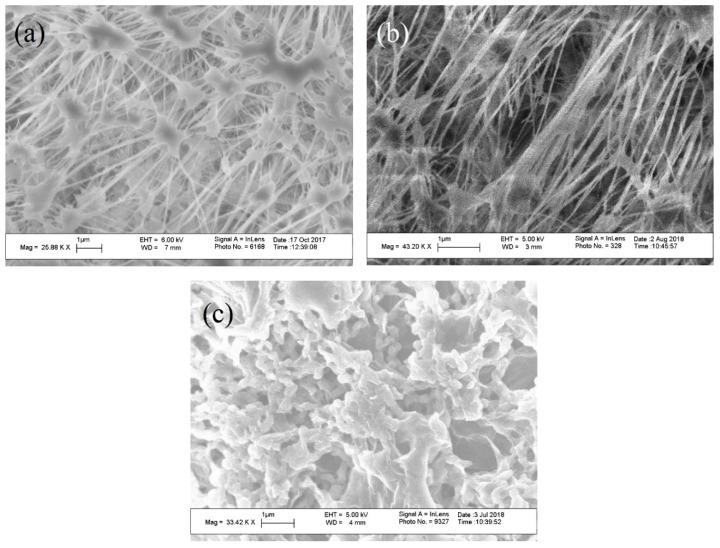
Scanning electron micrographs of (**a**) feed side and (**b**) permeate side of the unmodified polytetrafluoroethylene (PTFE) membrane; and, (**c**) graphene oxide immobilized membrane on the permeate side (GOIM-P) (permeate side).

Figure 2a–c show the SEM images of the unmodified PTFE membrane (feed side and permeate side), and GOIM-P (permeate side), respectively. Figure 2a clarifies the presence of active pores on the membrane feed surface. While Figure 2c demonstrates the change in morphology from Figure 2b due to the immobilization of the GO on the permeate surface.

**Figure 3:**
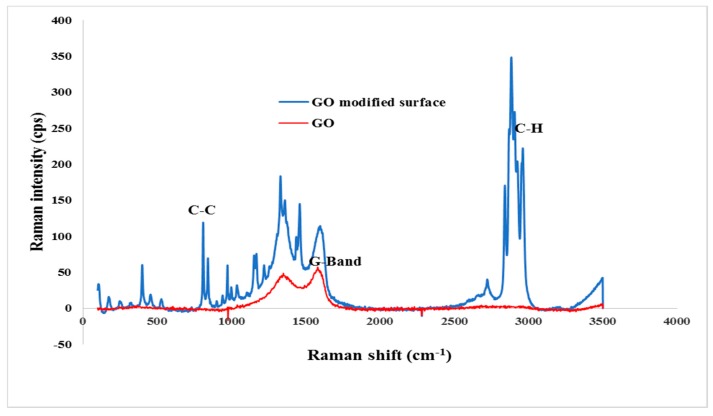
Raman spectra of the permeate surface of GOIM-P.

Raman spectra of the GOIM-P are shown in Figure 3. Prominent Raman peaks of support polypropylene layer on the permeate side of the composite membrane were observed at 800, 1500, 2700, and 3000 cm^−1^ [49]. The presence of GO on the membrane is shown at 1349 cm^−1^ that could be ascribed to the graphite defect in the sp3 domain via oxidation and an additional peak at 1597 cm^−1^ is due to stretching mode of graphite [50].

**Figure 4:**
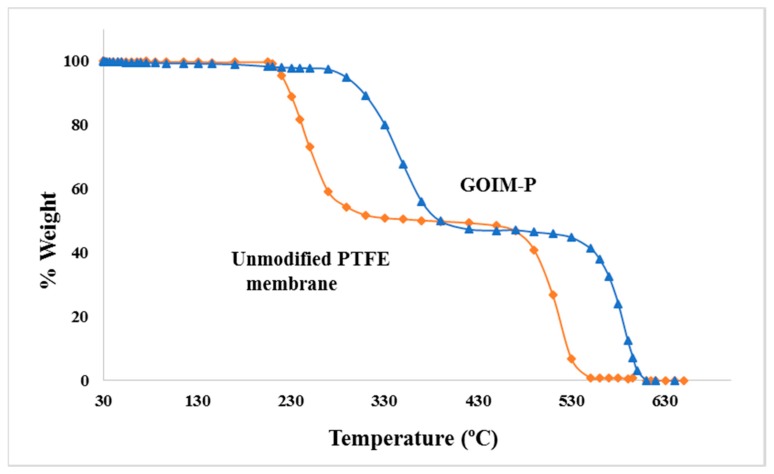
Thermal gravitational analysis (TGA) curves of unmodified and GOIM-P.

The thermal stability is an important parameter for membranes used in MD as the membranes must resist with high temperature salt solution. In this study, TGA curves were used to evaluate the stability of GOIM-P in comparison with unmodified membrane, as shown in Figure 4. The weight loss at around 230 °C to 330 °C was due to the decomposition of PP as the supporting layer, while PTFE began to decompose at around 460–470 °C. It was observed that the presence of GO provided additional thermal stability of the modified membrane. Increasing in the thermal stability of GOIM-P could be due to GO particles and the functional groups on GO that play the role as a reducing and sacrificed agent that lead to slow down or limit the degradation process [51]. The result is in line with what we have reported previously [32,36].

**Figure 5:**
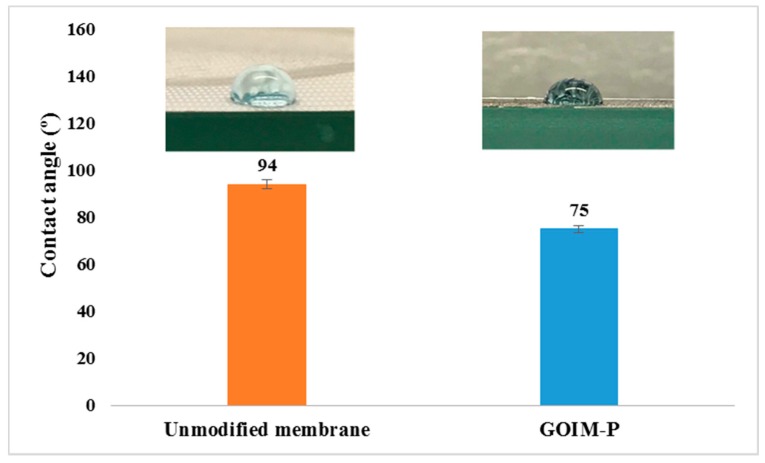
Contact angle and photographs of permeate side of unmodified and GOIM-P.

The hydrophobicity on the permeate surface of GOIM-P was determined by contact angle analysis. After GO immobilization, the contact angle on the permeate side of the modified membrane showed a decrease from 94° ± 2° to 75° ± 2°. A decreasing of contact angle implied that the hydrophilicity on the permeate side of the GOIM-P increases by increasing the surface energy and this was expected to enhance the membrane performances. The contact angle values and photographs of permeate side of unmodified and GOIM-P are shown in Figure 5.

### 3.2. DCMD Performance of GOIM-P

The overall permeate flux, $J_{w}$, is expressed as:

(1) $$J_{w}=\frac{W_{p}}{t\cdot A}\;$$

where, $W_{p}$ is the total mass collected from the permeate side, t is the run time, and A is the effective membrane area. Temperature, feed flow rate, and salt concentration were varied in the experiment to evaluate the performance of the GOIM-P, and of the unmodified membrane.

#### 3.2.1. Effect of Temperature and Feed Flow Rate on Water Vapor Flux

**Figure 6:**
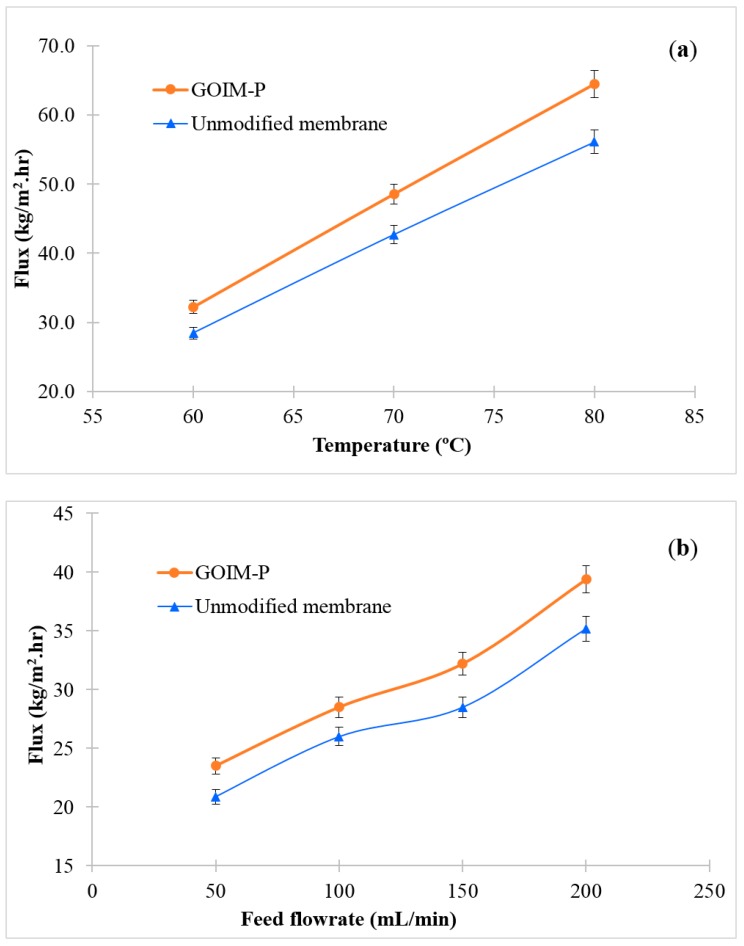
Effect of (**a**) temperature on water vapor flux at a feed flow rate of 150 mL/min; and, (**b**) feed flow rate on water vapor flux at feed temperature of 60 °C.

The effect of temperature on permeate flux of the GOIM-P when compared to the pristine membrane is illustrated in Figure 6a. It is clear from the figure that the fluxes significantly increased with increase in temperature for both membranes. Increasing in vapor pressure with temperature plays the major role in flux increment [52]. It is clearly seen that the GOIM-P produced a higher amount of permeated water when compared to the pristine membrane. Maximum permeate flux was 64.5 kg/m^2^·h at feed temperature of 80 °C, which is 15% higher as compared to the pristine membrane.

The influence of increasing feed flow rate at a constant temperature of 60 °C and 200 mL/min permeate flow rate is displayed in Figure 6b. It was observed that the permeate flux increased with an increase in feed flow rate in both membranes and the GOIM-P offered higher water vapor flux when compared to the unmodified one. The increased feed flow rate enhances the turbulence and reduces the boundary layer effect at the membrane-feed solution interface. These result in the reduction of temperature polarization and improving the permeate flux [53,54].

#### 3.2.2. Effect of Salt Concentration

**Figure 7:**
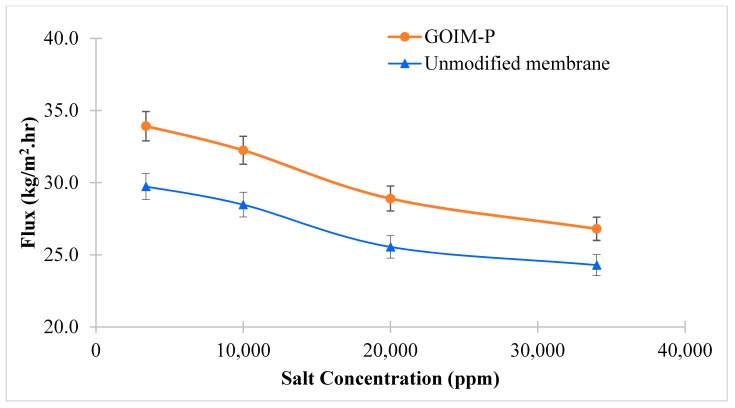
Effect of varying feed concentration on water vapor flux at feed flow rate of 150 mL/min and operating temperature of 60 °C.

Figure 7 shows the effect of varying salt concentrations in feed solution on permeate flux. With increase in concentration, both membranes show a decrease in water vapor flux as expected. The increase in feed salt concentration led to the reduction of the water activity at the membrane-solution interface and the formation of additional boundary layer directly affect the driving force across the membrane and reduces the water vapor flux. Similar results have been reported before [55,56]. As a result of increasing salt concentration from 3400 to 34,000 ppm, the permeate flux reduced from 29.7 to 24.3 kg/m^2^·h, and 33.9 to 26.8 kg/m^2^·h for the unmodified membrane and GOIM-P, respectively. The conductivity of the permeated water did not change with varying salt concentrations, indicating the complete rejection of the salt.

#### 3.2.3. Mass Transfer Coefficient

The overall, mass transfer coefficient can be described as:

$$\;J_{w}=k\left(P_{f}-P_{p}\right)$$

(2) $$\mathrm{or},\;k=\frac{J_{w}}{\left(P_{f}-P_{p}\right)}\;$$

where, $J_{w}$ is the water vapor flux, k is mass transfer coefficient, and $P_{f}$ and $P_{p}$ are partial vapor pressure of average feed and permeate temperatures. The mass transfer coefficients were found to be higher for GOIM-P as compared to the unmodified membrane.

Table 1 summarizes the change in mass transfer coefficients of GOIM-P and the unmodified membrane with varying feed flow rate at 60 °C. Both membranes exhibited increased mass transfer coefficient with increase in feed flow rate. The diffusion of the water vapor through the boundary layers mainly controls the overall mass transfer rate of the process. At higher feed flow rate, the turbulence increased that led to the reduction in the boundary layer resistance and significantly increased the mass transfer coefficients. Among these two membranes, GOIM-P exhibited higher mass transfer coefficient in comparison with the pristine membrane.

### 3.3. Stability and Salt Breakthrough

**Figure 8:**
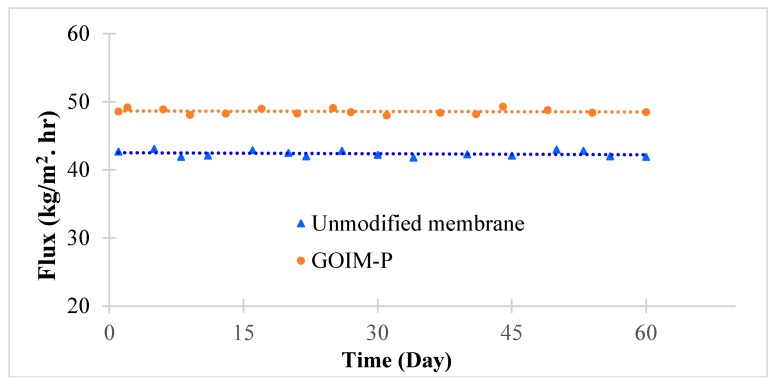
Stability of the membranes at a 70 °C feed temperature and 10,000 ppm of NaCl solution.

The quality of permeate side water was carefully investigated to monitor the stability of modified membrane and salt breakthrough. The stability of GOIM-P was tested for a long period of operation, as shown in Figure 8. The permeated water was monitored throughout the experiment to ensure the quality of water by measuring the conductivity of the permeate side water and using Raman spectroscopy [43,57]. The results did not show any leakage of salt through the membrane and the presence of GO in the permeate water samples.

## 4. Proposed Mechanism

**Figure 9:**
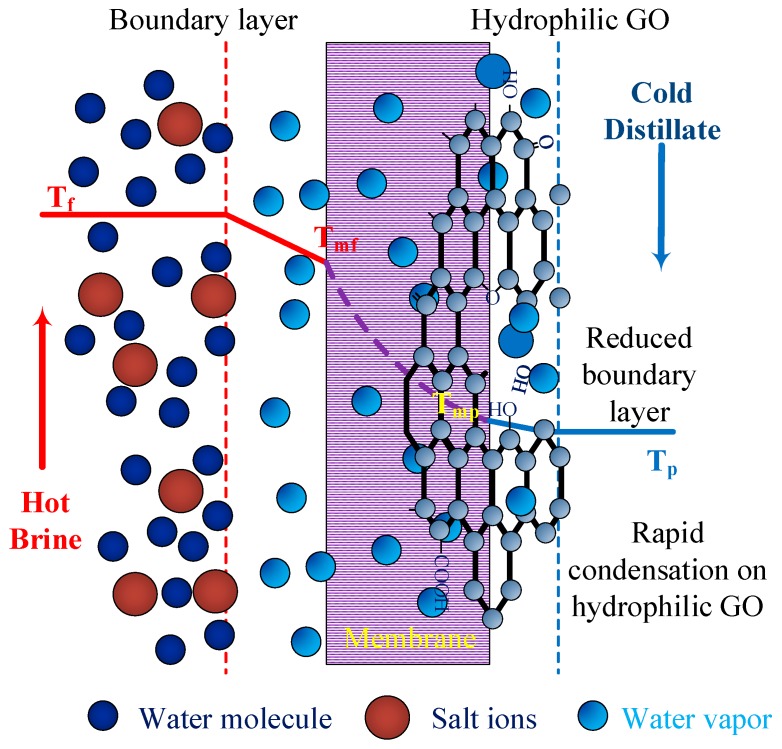
Proposed mechanism for GOIM-P.

The proposed mechanism in GOIM-P is shown in Figure 9. A significant enhancement in water vapor flux was noticed with the inclusion of GO in the permeate side. The hydrophilicity of the membrane permeate side was enhanced due to the presence of polar epoxy and carboxyl and hydroxyl functional groups on GO that allow for the water vapor to interact with the modified permeate surface [58,59]. In MD, the water vapor permeation through the membrane is steered by the vapor pressure gradient present across the membrane. A boundary layer compromising probably of both liquid and vapor phases is formed on both side of the membrane. Although, the feed side layer remains unchanged in GOIM-P, the hydrophilic surface permitted fast water vapor removal, destabilization of vapor-gap, and mass transfer resistance reduction between the bulk permeate and membrane surface. These effects are equivalent to the contraction in permeate side boundary layer [16,60,61], which led to an enhancement in water vapor permeation through the membrane.

## 5. Conclusions

GO was successfully immobilized on the permeate side of the PTFE membrane to increase the pure water flux in direct contact membrane distillation. It was evident that the introduction of hydrophilicity on the permeate side was effective in the rapid condensation and removal of the permeate, thus enhancing the mass transfer coefficient. The DCMD performance of GOIM-P was consistently superior compared to the pristine membrane and attaining a maximum water vapor flux of 64.5 kg/m^2^·h at 80 °C, which is 15% higher. The membrane was also found quite stable for a longer period of operation.

## Figures and Tables

**Table 1:** Effect of varying feed flow rate on mass transfer coefficient at 60 °C.

Feed Flow Rate (mL/min)	*k* (kg/m^2^·s·Pa) × 10^−7^	
	Unmodified Membrane	GOIMP
50	3.3	3.7
100	4.1	4.5
150	4.5	5.1
200	5.56	6.2
